# AKI in chronic myeloid leukemia: remember lysozyme-induced nephropathy

**DOI:** 10.1590/2175-8239-JBN-2024-0163en

**Published:** 2025-03-21

**Authors:** Adriana Santos, Nídia Marques, Roberto Pestana

**Affiliations:** 1 Unidade Local de Saúde São João, Departamento de Nefrologia, Porto, Portugal.; 2 Unidade Local de Saúde São João, Departamento de Patologia Anatômica, Porto, Portugal.

**Figure 1:**
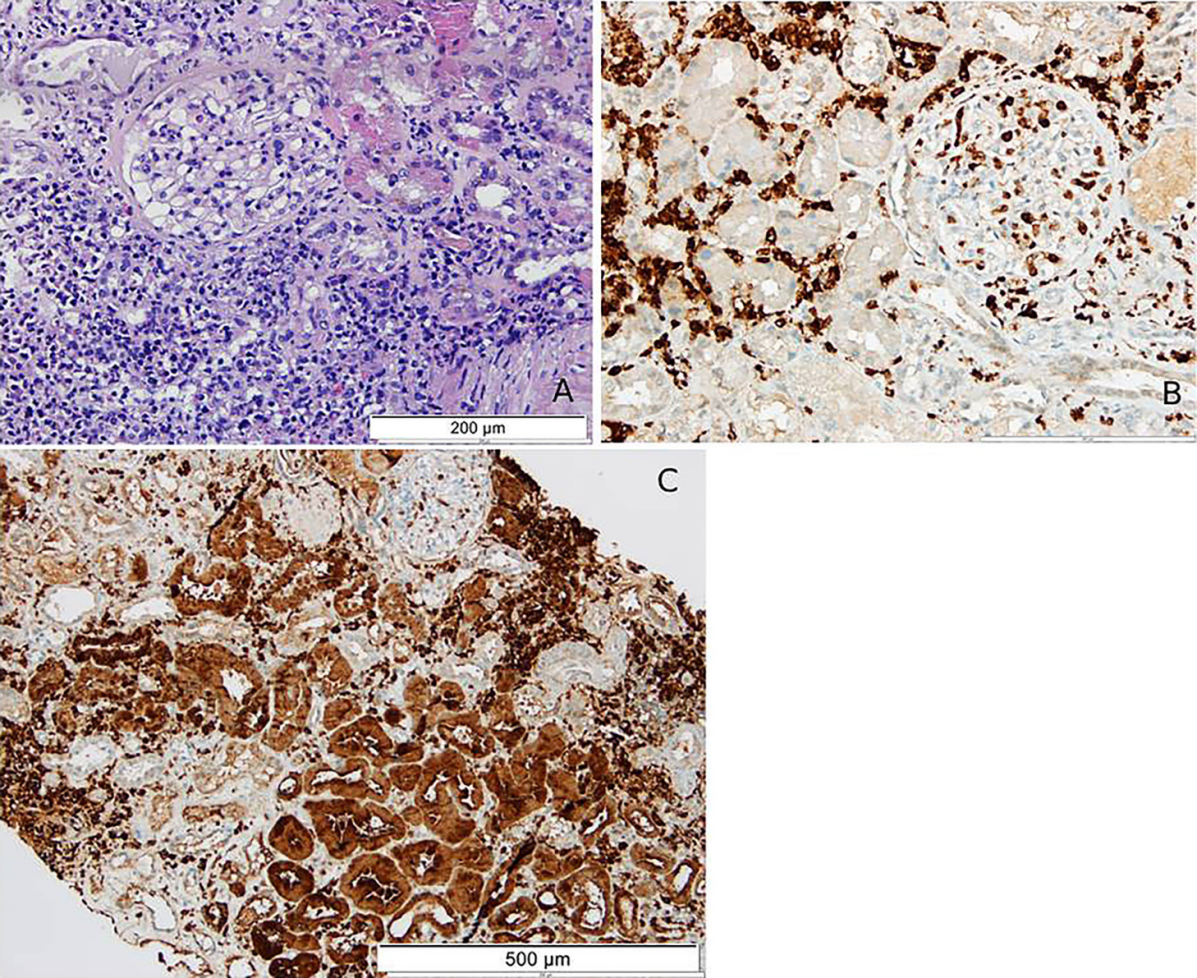
Renal cortex with myeloid precursors with signs of monocytic differentiation in the interstitium. (Figure 1A – Hematoxylin-Eosin staining, 200x). The immunohistochemical study displays myeloid cells positive for myeloperoxidase (Figure 1B – 200X). The proximal tubules and the myeloid cells were both positive for lysozyme (Figure 1C – 100x).

A 64-year-old woman with progressive atypical chronic myeloid leukemia awaiting an allogeneic bone marrow transplant was admitted to the hospital for acute kidney injury (AKI). Physical examination revealed peripheral edema. Blood tests revealed a serum creatinine of 3.65 mg/dL, urea of 133 mg/dL, and albumin of 23.9 g/L. Urinary sediment showed erythrocituria (1497/μL) and a 24-hour urine collection revealed proteinuria of 3.67 g (with albuminuria of 1.6g). Renal ultrasound ruled out obstruction. The patient’s condition deteriorated, leading to oliguria and requiring urgent hemodialysis. A renal biopsy was performed (Figure 1) and in addition to leukemic infiltration, lysozyme-induced nephropathy was diagnosed. This entity, usually associated with subnephrotic-range proteinuria, is a rare cause of AKI in patients with myelomonocytic leukemia and is associated with poor outcomes^1,2^.
