# Supplementary material for: AKI in chronic myeloid leukemia: remember lysozyme-induced nephropathy
Source: J Bras Nefrol. 2025 Mar 21;47(3):e20240163. doi: 10.1590/2175-8239-JBN-2024-0163en (PMC11927183; doi:10.1590/2175-8239-JBN-2024-0163en)
Supplement: Translation

# Translation: IRA na leucemia mieloide crônica: estejamos atentos à nefropatia induzida por lisozima

**DOI:** 10.1590/2175-8239-JBN-2024-0163pt

**Authors:** Adriana Santos, Nídia Marques, Roberto Pestana

**Affiliations:** 1 Unidade Local de Saúde São João, Departamento de Nefrologia, Porto, Portugal.; 2 Unidade Local de Saúde São João, Departamento de Patologia Anatômica, Porto, Portugal.

Uma mulher de 64 anos com leucemia mieloide crônica atípica progressiva, aguardando transplante alogênico de medula óssea, foi internada devido à injúria renal aguda (IRA). O exame físico revelou edema periférico. Os exames de sangue indicaram creatinina sérica de 3,65 mg/dL, ureia de 133 mg/dL e albumina de 23,9 g/L. O sedimento urinário apresentou eritrocitúria (1497/μL) e a coleta de urina de 24 horas revelou proteinúria de 3,67 g (com albuminúria de 1,6 g). A ultrassonografia renal descartou obstrução. A condição da paciente se deteriorou, levando à oligúria e à necessidade de hemodiálise urgente. Realizou-se biópsia renal (Figura 1) e, além da infiltração leucêmica, foi diagnosticada nefropatia induzida por lisozima. Essa entidade, geralmente associada a proteinúria em faixa subnefrótica, é uma causa rara de IRA em pacientes com leucemia mielomonocítica e está associada a desfechos desfavoráveis^1,2^.

**Figura 1.** Córtex renal com precursores mieloides com sinais de diferenciação monocítica no interstício. (Figura 1A – Coloração Hematoxilina-Eosina, 200x). O estudo imuno-histoquímico revela células mieloides positivas para mieloperoxidase (Figura 1B – 200X). Os túbulos proximais e as células mieloides foram positivos para lisozima (Figura 1C – 100x).
